# Neisseria Cinerea Bacteremia Secondary to a Retropharyngeal Abscess

**DOI:** 10.7759/cureus.14217

**Published:** 2021-03-31

**Authors:** Kaitlin McDade, Abhinav Singla, David Pash, Mary Bavaro, Colin De La Houssaye

**Affiliations:** 1 Internal Medicine, Skagit Regional Health, Mount Vernon, USA; 2 College of Osteopathic Medicine, Pacific Northwest University of Health Sciences, Yakima, USA; 3 Internal Medicine/Infectious Disease, Skagit Regional Health, Mount Vernon, USA

**Keywords:** neisseria cinerea, bacteremia, retropharyngeal abscess

## Abstract

*Neisseria cinerea* is a commensal bacteria of the human oropharynx, not thought to be pathogenic, and is rarely associated with serious infections, including bacteremia. Case reports involving invasive *N. cinerea *infections are uncommon in the literature. Retropharyngeal abscesses are unusual in adults, and are usually attributable to local trauma.Based on a review of the literature, *Neisseria cinerea* bacteremia secondary to a retropharyngeal abscess has not been described. We present a unique case of an elderly female without clear predisposing factors for a retropharyngeal abscess, who presented with a *N. cinerea* bacteremia and was found to have an asymptomatic retropharyngeal abscess.

## Introduction

*Neisseria cinerea* is a commensal bacteria of the human oropharynx, with low pathogenic potential, and very few reported cases of bacteremia [1]. Only a handful of case reports involving invasive *N. cinerea* infections are documented; and the affected patients had underlying comorbidities and immunosuppression [1-4]. Furthermore, retropharyngeal abscesses are rare in adults, and are usually attributable to local trauma, such as fishbone ingestion or instrumentation [5,6]. The classic presentation of a retropharyngeal abscess involves sore throat, fever, dysphagia, odynophagia and trismus. To our knowledge, there are no case reports describing *Neisseria cinerea* bacteremia secondary to a retropharyngeal abscess. Here we present a unique case of an elderly female without predisposing factors, who presented with fever and confusion, but lacked typical symptoms of a retropharyngeal abscess, which is suspected to be the source for her *N. cinerea* bacteremia. 

## Case presentation

We present the case of a 91-year-old female with a past medical history of hypertension, hypothyroidism and hypercholesterolemia, who presented to the emergency department of Skagit Valley Hospital in Mount Vernon, Washington with a chief complaint of abdominal pain with nausea, constipation, and generalized weakness. The night prior to presentation, she awoke suddenly with subjective fever and rigors which resolved spontaneously by the following morning. Her daughter reported that the patient was confused, but is mentally sharp at baseline. In the Emergency Department, vital signs were significant for a temperature of 38.7 degree C, blood pressure of 83/43 with oxygen saturation of 95% on room air. Her temperature increased to 39.5C in the emergency department. Physical examination was significant for mild diffuse abdominal tenderness to palpation and mild confusion. She was started on norepinephrine in the emergency department, as well as piperacillin-tazobactam and azithromycin for suspected pneumonia. CT scan of abdomen and pelvis with contrast was notable for a small bilateral opacification at the lung bases (L>R), with an incidental finding of a 3.7 x 3.2cm distal abdominal aortic aneurysm. 

**Figure 1:**
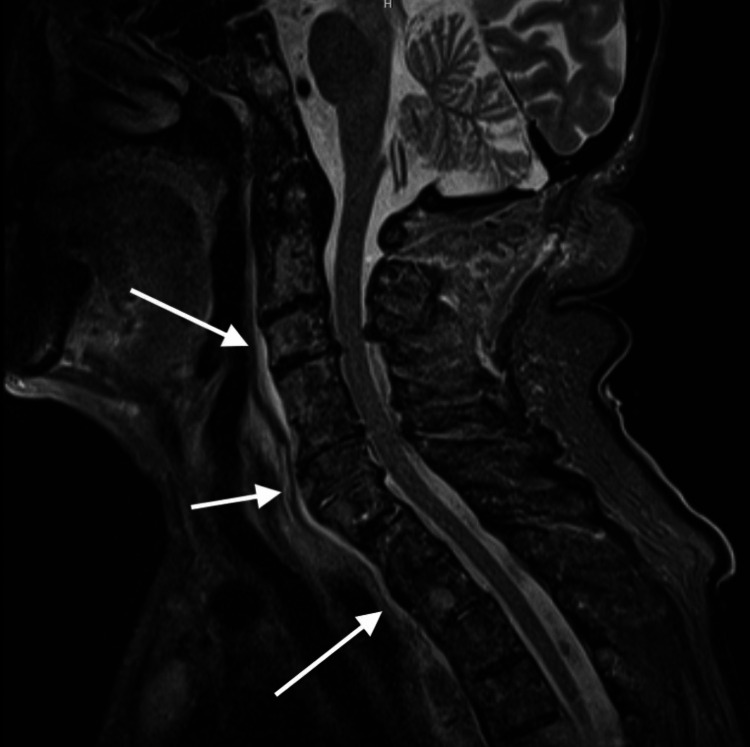
Sagittal view of retropharyngeal abscess, spanning from C2-T4 with maximum AP thickness of 7 mm.

Admission laboratory examinations were significant for WBC 3.5 x 10e3/uL, LDH 257 U/L, lactate 3.2 mmol/L and procalcitonin 31.20 ng/mL. SARS-CoV-2 was negative. Blood cultures were obtained while in the emergency department. On the second hospital day, she was afebrile and norepinephrine was discontinued. Blood cultures grew *Neisseria cinerea *in two out of four bottles. The patient’s lactate normalized and procalcitonin decreased to 27.97 ng/mL. Symptomatically, her abdominal pain resolved; however, she developed neck pain and headache, without meningeal signs. Based on the positive blood cultures, azithromycin was discontinued on hospital day 3. On hospital day 3, her WBC normalized to 6.4x10e3/uL, and she continued to be afebrile and normotensive, however her head and neck pain remained constant. Repeat blood cultures remained negative. On hospital day 4, due to unclear source of infection and ongoing neck pain, an MRI of the head and neck was obtained. This demonstrated a 7 mm abnormal fluid collection in the retropharyngeal space with surrounding soft tissue edema (Figure 1). These findings were concerning for a retropharyngeal abscess, with possible involvement of the danger space. Her head and neck pain had completely resolved but based on MRI findings, she was evaluated by ENT, who recommended medical management without the need for surgical drainage. On hospital day 5, piperacillin-tazobactam was discontinued and she was started on ceftriaxone and oral metronidazole. She was discharged on hospital day 6, with a treatment plan to continue oral metronidazole 500 mg three times daily and ceftriaxone 2 g daily pending further follow-up in the Infectious Diseases clinic with serial imaging to document resolution of the abscess. 

## Discussion

Odontogenic infections are often polymicrobial in nature, and can contain both anaerobic and aerobic organisms [6].

*Neisseria cinerea* is an aerobic, asaccharolytic, gram-negative, oxidase-positive, catalase-positive diplococcus [4]. The susceptibility of N. cinerea to colistin, its ability to grow on tryptic soy or Mueller-Hinton agar, and its inability to grow on modified Thayer-Martin medium help differentiate it from gonococci [7], as it has been mistaken for both *N. gonorrhaeae* and *B. catarrhalis*.

 To our knowledge, there are limited reports of extensive infections secondary to *Neisseria cinerea*, and only a handful of *Neisseria cinerea *bacteremia cases reported. The cases of *Neisseria cinerea* bacteremia include one case of posttraumatic meningitis with bacteremia, bacteremia in one post-splenectomy patient, one patient with AIDS, and one case of endocarditis in an intravenous drug user. Other cases of infections involving *Neisseria cinerea* include two patients with continuous ambulatory peritoneal dialysis (CAPD)-associated peritonitis, two cases of pulmonary infections in immunosuppressed patients, one case of proctitis in a child, and several cases of purulent conjunctivitis in neonates [1-5].

Retropharyngeal abscesses are uncommon in adults, and are typically secondary to local trauma such as fishbone ingestion, instrumentation, or dental infection, and like many other conditions, those with comorbidities are more susceptible. Of a study conducted in Germany to evaluate the prevalence of deep neck infections in adults, only 21.6% were older than 65 years of age (average age 73) [8]. Among these, 45% had an underlying comorbidity, with the most common being diabetes. In the elderly age group, the most common presenting symptoms were sore throat, odynophagia and neck pain [8]. While our patient did develop neck pain in the hospital, this was not present on admission. She presented only with symptoms of a resolved fever, abdominal pain, and mild confusion. She also lacked the typical physical exam findings of swelling of the oropharynx, or bulging of the posterior pharyngeal wall. In the same study, the most common source of infection was odontogenic, followed by tonsillar and salivary gland [8]. Our patient did not have any clear source of dental or tonsillar infection. Deep neck space infections are typically polymicrobial, with the most common isolates being viridans streptococci and *Streptococcus anginosus*, and anaerobic species present in dental infections. In otogenic infections, staphylococcus and pseudomonas must also be considered, while *Streptococcus pneumoniae, Haemophilus influenzae* and *Moraxella catarrhalis* are present in sinogenic infections [5]. While *N. cinerea *can be a commensal bacteria of the human oropharynx, with low pathogenic potential, it is on occasion pathogenic, as was seen in this case. 

The etiology of this patient’s bacteremia was postulated to be related to the retropharyngeal abscess, as no other clear source was identified. The question arose of whether our patient experienced either a transient bacteremia associated with the abscess, or from her mouth or gastrointestinal tract, as her blood cultures cleared quickly, thus potentially reflecting a very low inoculum. Our patient was admitted on azithromycin and piperacillin-tazobactam to cover for a suspected community-acquired pneumonia. She remained on piperacillin-tazobactam when her blood cultures returned positive, as *N. cinerea* is typically susceptible to ceftriaxone, penicillin, ampicillin and ciprofloxacin. This was continued during her hospitalization to cover both the *N. cinerea*, as well as a likely polymicrobial retropharyngeal abscess. Her treatment plan on discharge was to continue intravenous ceftriaxone and a brief course of metronidazole for additional anaerobic coverage. 

## Conclusions

In summary, this case of a 91-year-old female without significant medical history represents a unique case of* Neisseria cinerea* bacteremia in a patient with an atypical presentation of a retropharyngeal abscess, which is an uncommon condition in her age group. As outlined above, there are only a few known cases of invasive* N. cinerea *infection, and no known cases to our knowledge of *N. cinerea* bacteremia secondary to a retropharyngeal abscess. Hence, it is important to recognize unusual organisms isolated from blood cultures and identify potential sources, given that sometimes less pathogenic organisms such as *N. cinerea* can be associated with serious infections such as head and neck abscesses. Identification of a source of bacteremia is important in order to develop an early and effective diagnosis and treatment plan and avoid further clinical complications.
